# Enhanced prognosis and regional cooperative rescue systems for acute myocardial infarction: insights from chest pain centers in Ningxia, China

**DOI:** 10.1007/s11739-025-03962-y

**Published:** 2025-05-20

**Authors:** Mohan Wang, Peng Wu, Juan Ma, Xueping Ma, Na Yang, Shaobin Jia, Ning Yan

**Affiliations:** 1 The First Clinical College of Ningxia Medical University, Yinchuan, 750004 China; 2 Heart Centre and Department of Cardiovascular Diseases, General Hospital of Ningxia Medical University, Yinchuan, 750004 China; 3 Institute of Medical Sciences, General Hospital of Ningxia Medical University, Yinchuan, 750004 China; 4 National Health Commission Key Laboratory of Metabolic Cardiovascular Diseases Research, Ningxia Medical University, Yinchuan, 750004 China; 5 Ningxia Key Laboratory of Vascular Injury and Repair Research, Ningxia Medical University, Yinchuan, 750004 China

**Keywords:** Chest pain center, Acute ST-segment elevation myocardial infarction, Reperfusion therapy, Prognosis

## Abstract

Chest Pain Centers (CPC) demonstrated improved outcomes for patients with acute myocardial infarction (AMI) globally. However, the long-term impact of CPC establishment in economically developing areas, such as Ningxia, China, remains unclear. This study aimed to assess the long-term prognosis and efficacy of collaborative regional rescue systems centered on CPC for ST-segment elevation myocardial infarction (STEMI) patients in Ningxia. This retrospective cohort study analyzed 5344 STEMI patients from the Ningxia Myocardial Infarction Registry (2014–2019). Based on CPC establishment, patients were segregated into two groups: pre-CPC (n = 2141) and post-CPC (n = 3203). Kaplan–Meier survival analysis and Cox proportional hazards models were employed to compare the groups and evaluate long-term outcomes, including mortality and major adverse cardiovascular and cerebrovascular events (MACCEs). A total of 5344 acute STEMI patients were included, with 2141 (40.06%) in the pre-CPC group and 3203 (59.94%) in the post-CPC group. In comparison to the pre-CPC group, the post-CPC group exhibited lower all-cause mortality rates at 30 days (4.53% vs. 6.68%, *p* = 0.001), 1 year (6.24% vs. 9.11%, *p* = 0.001), and 3 years (8.55% vs. 11.86%, *p* < 0.001). Additionally, the post-CPC group showed decreased rates of MACCEs at 30 days (7.90% vs. 10.00%, *p* = 0.008) and 3 years (18.86% vs. 23.12%, *p* < 0.001). Kaplan–Meier survival analysis yielded similar results. After adjusting for confounding factors using COX multivariable regression, the CPC establishment was found to be a protective factor for all-cause mortality and MACCEs within 30 days (MACCEs: HR = 0.72, 95%CI 0.59–0.88,* p* = 0.005; all-cause mortality: HR = 0.59, 95%CI 0.46–0.77,* p* < 0.001), 1 year (MACCEs events: HR = 0.80, 95%CI 0.68–0.94, *p* = 0.006*;* all-cause mortality: HR = 0.59, 95%CI 0.44–0.69,* p* < 0.001), and 3 years (MACCEs: HR = 0.71, 95%CI 0.62–0.81,* p* < 0.001; all-cause mortality: HR = 0.55, 95%CI 0.46–0.67,* p* < 0.001). The establishment of Chest Pain Centers and implementation of regional cooperative rescue systems significantly improved the long-term prognosis of STEMI patients in Ningxia. These findings underscore the importance of developing CPC in underdeveloped regions to enhance cardiovascular emergency care and reduce mortality and morbidity associated with acute myocardial infarction.

## Introduction

Cardiovascular diseases (CVDs) remain the leading cause of death worldwide, accounting for over 18 million deaths annually according to the Global Burden of Disease (GBD) study [1]. In China, ischemic heart disease (IHD) has emerged as the primary contributor to CVD-related mortality, with a doubling in death rates over the past two decades [2]. Currently, over one million lives are lost annually to IHD in China, surpassing other major non-communicable diseases [3]. This growing burden is further exacerbated by rapid urbanization, Westernized lifestyles, and a decline in physical activity, which have collectively fueled the rise of CVDs [4, 5]. The hospitalization rate for ST-segment elevation myocardial infarction (STEMI) in China increased nearly fourfold between 2001 and 2011[4]. However, despite advances in reperfusion therapy and pharmacological interventions, in-hospital mortality rates for STEMI have not significantly improved [6]. Notably, since 2013, the incidence of acute myocardial infarction (AMI) in rural areas has surpassed urban rates, highlighting disparities in access to quality care [7]. Compared to the United States and Europe, where STEMI management has seen substantial advancements, China’s capacity to treat acute myocardial infarction remains in urgent need of improvement [8, 9]. Given the persistently severe trends in CVD morbidity and mortality, strengthening STEMI treatment infrastructure and implementing comprehensive prevention strategies are critical for reversing the growing burden of CVD in China.

STEMI is one of the most severe and significant types of cardiovascular disease, resulting from acute coronary artery occlusion [10]. However, the prevention and management of STEMI remain inadequate in many regions due to several key factors. These include delays in seeking medical attention, insufficient awareness of early STEMI symptoms among the public, inadequate pre-hospital emergency systems, and disparities in healthcare infrastructure that hinder access to timely reperfusion therapy, such as primary percutaneous coronary intervention (PCI) [11, 12]. Additionally, variability in the adoption of guideline-directed medical therapy and a lack of standardized treatment protocols further contribute to poor outcomes. In response to these challenges, many countries have implemented Chest Pain Centers (CPC) to optimize STEMI care by streamlining early diagnosis, improving access to reperfusion therapies, and ensuring adherence to evidence-based treatment strategies [13, 14]. Several studies from various countries have consistently demonstrated that the establishment of CPC is significantly associated with better performance on core quality measures, suggesting that these improvements may also be linked to enhanced outcomes for acute myocardial infarction patients [15–19]. While previous studies have highlighted the potential benefits of CPC in improving in-hospital outcomes for acute STEMI, there is limited evidence on their long-term impact on patient prognosis and survival rates beyond the hospital discharge period. Particularly in Northwest China, economically underdeveloped regions still lack sufficient evidence regarding treatment outcomes.

The Ningxia Hui Autonomous Region, located in China’s economically underdeveloped northwestern inland region, faces significant challenges in healthcare access and resource allocation. To enhance STEMI management and improve patient outcomes, the General Hospital of Ningxia Medical University has actively pursued the establishment of a Chest Pain Center. Located in the capital city of Yinchuan, the General Hospital of Ningxia Medical University represents a key healthcare institution in the region. In November 2016, it became one of the first hospitals in China to receive certification as a CPC and has since been identified as a key development site for the National Regional Medical Center. The hospital’s mission is to establish a rapid response mechanism for chest pain management, thereby improving the efficiency of acute cardiovascular disease care and reducing mortality associated with chest pain and related conditions. As a regional hub for chest pain management, the center serves not only Yinchuan but also nearby counties, cities, and even neighboring provinces such as Gansu and Shaanxi. This broad service coverage enhances access to timely and effective care for patients in both urban and rural areas. Furthermore, the CPC facilitates medical collaboration and patient referral systems with other healthcare institutions in the region, including hospitals in surrounding cities and counties. These partnerships contribute to raising the overall standard of care in the region, particularly in the field of cardiac emergency services.

By addressing critical gaps in acute cardiovascular care and strengthening the regional healthcare system, the CPC plays an integral role in reducing the burden of cardiovascular diseases in northwestern China. Through these efforts, it actively advances the STEMI treatment capabilities of the Ningxia region, providing essential care to a population in need. However, this region continues to face significant challenges in the prevention and treatment of myocardial infarction, including uneven allocation of medical resources and a relative shortage of high-quality healthcare services. According to the China Cardiovascular Health Index (2017), the cardiovascular health index score for Ningxia was only 47.4 points, with a particularly low score of 27.11 points for cardiovascular disease treatment capabilities and outcomes [20]. These scores ranked among the lowest in the nation, underscoring the severe challenges in cardiovascular disease prevention, treatment, and control in this region. These findings highlight the pressing need to address deficiencies in cardiovascular care.

In response, a comprehensive clinical registry study was launched to identify and address clinical issues and challenges specific to acute myocardial infarction care in Ningxia. The study seeks to establish improved diagnostic and treatment protocols tailored to the region’s unique circumstances. Its primary objective is to compare the care and outcomes of patients hospitalized for STEMI before and after the establishment of the CPC.

## Methods

### Study design and population

The study was designed in strict accordance with the Strengthening the Reporting of Observational Studies in Epidemiology (STROBE) guidelines. This study complied with the Declaration of Helsinki and was approved by the Ethics Committee of the General Hospital of Ningxia Medical University (approval number 2020–774). Given this was a retrospective cohort, follow-up was carried out via telephone, with the follow-up period ending in January 2023. Written informed consent was obtained from individuals for the publication of any potentially identifiable images or data included in this study. This study has been registered on the Chinese Clinical Trials Registry (registration number: ChiCTR2100043359).

**Fig. 1:**
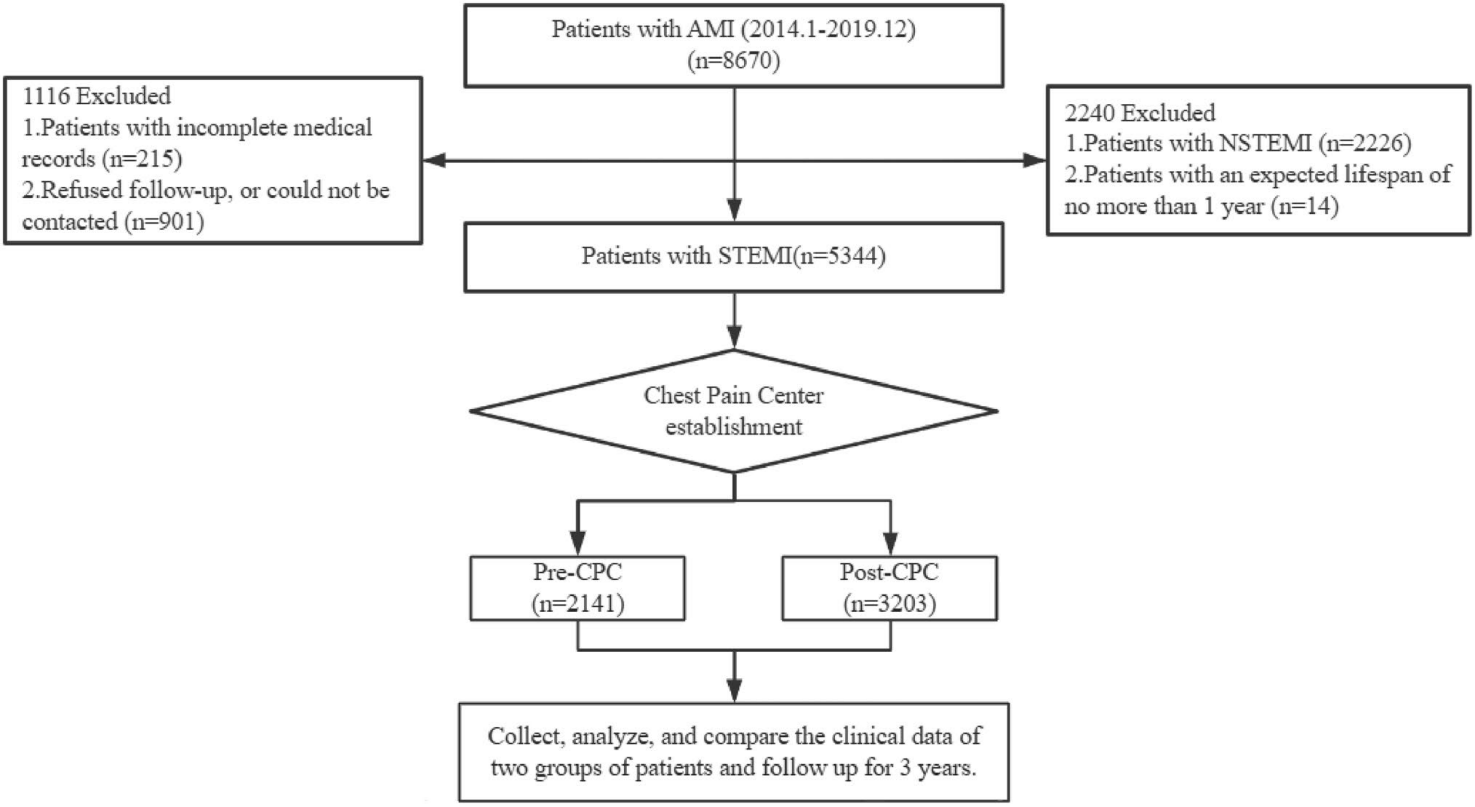
The flow chart of research subject. *CPC* chest pain center, *AMI* acute myocardial infarction, *STEMI* ST-segment elevated myocardial infarction, *NSTEMI* non- ST-segment elevated myocardial infarction

This study was designed as a single-center, retrospective cohort investigation. The registry includes patients admitted with AMI to the General Hospital of Ningxia Medical University between January 2014 and December 2019. The patient flow chart is shown in Fig. 1. The initial cohort consisted of 8670 AMI patients. All participants met European Society of Cardiology guidelines for the diagnosis and treatment of STEMI [11]. Inclusion: (1) AMI is defined as when there is evidence of myocardial injury (defined as an elevation of cardiac troponin values with at least one value above the 99 th percentile upper reference limit) with necrosis in clinical setting consistent with myocardial ischemia; (2) ST-segment elevation in at least two contiguous leads as STEMI; (3) Primary PCI means that patient has PCI procedure within 12 h of the onset of symptoms. Exclusion: (1) Non-STEMI (NSTEMI) patients (2226 cases); (2) Incomplete medical records (215 cases); (3) Patients with follow-up durations less than one year (14 cases); (4) Refused follow-up or could not be contacted (901 cases). Finally, 5344 eligible patients were enrolled in this study and were categorized into two groups based on the timing of chest pain center establishment: the pre-CPC group (n = 2141) and the post-CPC group (n = 3203).

## Intervention: chest pain center protocol

The Chest Pain Center at General Hospital of Ningxia Medical University became accredited by the China Chest Pain Center Headquarters in November 2016. It implements a structured protocol to expedite diagnosis and intervention for individuals experiencing acute chest pain [21].

Chest Pain Centers (CPCs) are specialized hospital-based networks designed to standardize and optimize the diagnosis and treatment of acute coronary syndromes (ACS), particularly STEMI. The establishment of CPCs in China follows the China Chest Pain Center Certification Standards, which emphasize regional cooperation, rapid diagnosis, and timely reperfusion therapy to reduce delays in STEMI management.

A CPC typically consists of the following key components:

1. Pre-hospital Emergency Network: CPCs collaborate with local emergency medical services (EMS) to establish a direct referral system. This enables pre-hospital electrocardiogram (ECG) acquisition, early STEMI identification, and activation of the catheterization lab before hospital arrival.
2. Multidisciplinary In-Hospital Coordination: CPCs integrate emergency departments (EDs), cardiology departments, catheterization labs, and intensive care units (ICUs) to ensure seamless communication and rapid decision-making for STEMI patients.
3. Standardized STEMI Diagnosis and Treatment Protocols: CPCs follow evidence-based guidelines, including early ECG acquisition (within 10 min of arrival), risk stratification, guideline-directed medical therapy (GDMT), and primary percutaneous coronary intervention (PCI) within 90 min of first medical contact (FMC).
4. Quality Control and Data Monitoring: CPCs maintain continuous quality assessment through real-time data collection on key performance indicators (e.g., door-to-balloon (D2B) time, thrombolysis-to-PCI conversion rates, adherence to medication protocols). The China Chest Pain Center Headquarters conducts regular audits to ensure compliance with national CPC standards.

The primary role of CPCs in STEMI care is to improve time-sensitive interventions and patient outcomes through the following strategies: Reducing pre-hospital and in-hospital delays by establishing a streamlined emergency response system; Enhancing the use of guideline-directed medical therapy (GDMT), including dual antiplatelet therapy, statins, and β-blockers; Optimizing resource allocation to ensure timely primary PCI for eligible patients and early fibrinolysis for those in non-PCI-capable hospitals; Providing post-discharge care to improve long-term secondary prevention and reduce STEMI recurrence and mortality.

### Data collection

Patient demographics, the clinical history, laboratory indicators, and data related to echocardiography and angiography were collected through the electronic medical record system. Demographic characteristics included age, gender, weight, height [to calculate body mass index (BMI)], heart rate (HR) and smoking history. Clinical history included established diabetes, hypertension, stroke, cerebrovascular disease, peripheral vascular disease, atrial fibrillation, coronary artery disease history, chronic kidney disease history, chronic obstructive pulmonary disease (COPD), gastrointestinal ulcer history, Killip classification.

Peripheral venous blood samples were collected from all patients within 24 h of admission in the early morning after overnight fasting for routine blood tests and biochemical analyses. The fully automated hematology analyzer XN-9000 (Sysmex, Japan) was applied to determine white blood cell count (WBC), hemoglobin (Hb), platelet count (PLT). Aspartate aminotransferase (AST), serum creatinine (SCR), total cholesterol (TC), triglycerides (TG), low-density lipoprotein cholesterol (LDL-C), and high-density lipoprotein cholesterol (HDL-C), were measured with a fully automated biochemical analyzer Atellica IM1300 (siemens, Germany). All patients underwent echocardiography within 24 h of admission by a specialized sonographer applying an GEvivid7 (GE HealthCare, USA) color Doppler ultrasound diagnostic instrument to measure left ventricular ejection fraction (LVEF).

The angiographic data was obtained from the cardiac catheterization laboratory records. Including the operative approach, dominant type, number of diseased vessels, location of target lesions (left main coronary artery (LM), left anterior descending (LAD), left circumflex artery (LCX), and right coronary artery (RCA). Besides that, information about medications (aspirin, clopidogrel, statins, beta blockers, angiotensin-converting enzyme inhibitors (ACEI)/angiotensin receptor Blocker (ARB) obtained. All data were collected retrospectively using a standardized data collection form. The clinical quality measures are included in the section on acute ST-segment elevation myocardial infarction in the Chinese Cardiovascular Disease-Related Professional Medical Quality Control Indicators (2021 Edition) [22].

### Follow‑up and endpoints

The follow-up period for this study was designed to evaluate the long-term outcomes of patients with ST-segment elevation myocardial infarction (STEMI) after their initial hospital treatment. Follow-up assessments were conducted at three key time points: initial follow-up (30 days), intermediate follow-up (1 year), and long-term follow-up (3 years) after the onset of STEMI. The follow-up process aimed to capture both short-term and long-term clinical outcomes, specifically focusing on major adverse cardiovascular and cerebrovascular events (MACCEs) and all-cause mortality.

The primary endpoint was the occurrence of MACCEs at three time points: 30 days, 1 year, and 3 years, encompassing all-cause mortality, nonfatal myocardial infarction, rehospitalization for angina, and nonfatal stroke. Follow-up data were obtained by trained personnel who conducted telephone interviews with patients or their family members. All follow-up information was cross-verified with hospital records to ensure accuracy. Between May 2023 and August 2023, telephone follow-up was successfully completed for 5344 patients, all of whom provided verbal consent to participate.

### Statistical analysis

The database for this study was established using the Epidata platform. Statistical analysis was conducted using R software version 4.2.3. For normally distributed data, values were expressed as the mean ± standard deviation (x ± s), and comparisons between groups were performed using independent sample t-tests. Non-normally distributed continuous variables were presented as the median and interquartile range (IQR), and differences between groups were analyzed using the Mann–Whitney U test. Categorical variables were expressed as counts and proportions, with comparisons between groups performed using the chi-squared test. Kaplan–Meier survival curves were used to evaluate time-to-event outcomes, and differences between survival curves were assessed using the Log-rank test. Multivariate Cox regression analysis was applied to investigate long-term clinical outcomes, with adjustments made for confounding factors. Variables included in the model were selected based on clinical relevance and statistical significance, ensuring a comprehensive adjustment for baseline differences. Variables included in the multivariable model were those demonstrating clinical or statistical significance, including age, sex, BMI, diabetes, hypertension, cerebrovascular disease, peripheral vascular disease, atrial fibrillation, COPD, gastrointestinal ulcer history, troponin-T, RBC, platelet, albumin, creatinine, sodium, chlorine, uric acid, ALT, creatine kinase, LDL cholesterol, HDL cholesterol, triglycerides, SBP at admission, DBP at admission, multivessel disease, Killip classification and no-reflow. We used a stepwise selection approach, ensuring that key demographic, clinical, and laboratory variables known to influence STEMI prognosis were included in the model. Results were presented as adjusted hazard ratios (HRs) with 95% confidence intervals (CIs). Statistical significance was set at a two-sided *p*-value of < 0.05 for all tests.

## Results

A total of 5344 patients were included, with 2141 in the pre-CPC group and 3203 in the post-CPC group. As shown in Table 1, the post-CPC group had slightly older patients (mean age: 61 vs. 60 years, *p* = 0.035) and higher rates of hypertension (50.86% vs. 48.01%, *p* = 0.042), diabetes (23.23% vs. 20.46%, *p* = 0.017), and prior stroke (11.27% vs. 9.11%, *p* = 0.011). Patients in the post-CPC group were more likely to present with less severe heart failure, as reflected by a higher proportion in Killip grade I (76.46% vs. 74.59%, *p* < 0.001). Blood pressure at admission improved significantly, with higher systolic (122.09 vs. 118.29 mmHg, *p* < 0.001) and diastolic pressures (76.36 vs. 74.53 mmHg, *p* < 0.001), indicating potential improvements in early myocardial injury management.

**Table 1:** Baseline characteristics of STEMI patients stratified by CPC establishment timing

Variable	pre-CPC (n = 2141)	post-CPC (n = 3203)	*P* value
Male (%)	1700 (79.40)	2531 (79.02)	0.736
Age (years)	60 (51–69)	61 (52–69)	0.035
BMI (kg/m^2^)	24.76 ± 3.34	24.61 ± 3.51	0.126
Diabetes history (%)	438 (20.46)	744 (23.23)	0.017
Hypertension history (%)	1028 (48.01)	1629 (50.86)	0.042
Cerebrovascular disease history (%)	195 (9.11)	361 (11.27))	0.011
Peripheral vascular disease history (%)	107 (5.00)	82 (2.56)	< 0.001
Coronary artery disease history (%)	218 (10.18)	347 (10.83)	0.448
Atrial fibrillation history (%)	22 (1.03)	66 (2.06)	0.004
Chronic obstructive pulmonary disease (%)	34 (1.59)	94 (2.93)	0.002
Chronic kidney disease history (%)	62 (2.90)	100 (3.12)	0.636
Gastrointestinal ulcer history (%)	70 (3.27)	65 (2.03)	0.005
Current smoking (%)	1336 (62.40)	1957 (61.19)	0.416
Killip classification (%)			< 0.001
I	1597 (74.59)	2449 (76.46)	
II	357 (16.67)	420 (13.11)	
III	80 (3.74)	79 (2.47)	
IV	107 (5.00)	255 (7.96)	
HR at admission (bpm)	80.02 ± 16.53	80.61 ± 16.13	0.197
SBP at admission (mmHg)	118.29 ± 22.59	122.09 ± 26.51	< 0.001
DBP at admission (mmHg)	74.53 ± 17.85	76.36 ± 18.12	< 0.001
Troponin-T (ng/ml)	17.40 ± 12.39	21.09 ± 22.23	< 0.001
WBC, 10^9/L	10.51 ± 3.60	10.67 ± 3.53	0.108
RBC, 10^9/L	4.62 ± 0.53	4.69 ± 0.58	< 0.001
Platelet count (10⁹/L)	219.91 ± 60.68	227.45 ± 67.49	< 0.001
Albumin (g/L)	37.68 ± 4.39	38.25 ± 4.23	< 0.001
Creatinine (μmol/L)	77.89 ± 39.62	74.81 ± 37.15	0.004
Potassium (mmol/L)	3.92 ± 0.42	3.90 ± 0.49	0.205
Sodium (mmol/L)	139.00 ± 4.76	139.58 ± 3.78	< 0.001
Chlorine (mmol/L)	105.44 ± 4.16	106.22 ± 3.77	< 0.001
Uric acid (µmol/L)	336.76 ± 93.83	342.67 ± 94.02	0.024
Glucose (mmol/L)	7.93 ± 3.57	7.83 ± 3.49	0.289
ALT (U/L)	78.16 ± 264.28	65.95 ± 104.48	0.042
AST (U/L)	182.87 ± 383.27	176.86 ± 205.55	0.507
CK (U/L)	1108.8 ± 1410.07	1276.19 ± 1568.56	< 0.001
Total cholesterol (mmol/L)	4.16 ± 0.94	4.14 ± 0.92	0.415
LDL-C (mmol/L)	2.28 ± 0.61	2.16 ± 0.58	< 0.001
HDL-C (mmol/L)	0.92 ± 0.18	0.93 ± 0.19	0.023
Triglycerides (mmol/L)	1.74 ± 1.11	1.58 ± 1.17	< 0.001
Left ventricular ejection fraction (%)	51.52 ± 10.03	50.97 ± 10.04	< 0.001
Multivessel disease (%)			< 0.001
0	33 (1.77)	39 (1.31)	
1	392 (21.01)	437 (14.70)	
2	575 (30.81)	839 (28.22)	
3	866 (46.41)	1,658 (55.77)	
IRA location (%)			
LM	7 (0.38)	25 (0.84)	0.052
LAD	974 (52.20)	1,430 (48.10)	0.006
LCX	205 (10.99)	358 (12.04)	0.265
RCA	661 (32.74)	986 (33.17)	0.762
No-reflow (%)	101 (5.41)	121 (4.07)	0.092
IABP insertion (%)	84 (4.50)	110 (3.70)	0.349
Hospital stay (days)	9.51 ± 4.86	8.80 ± 4.22	< 0.001
In-hospital cost (RMB, in thousands)	52.64 ± 32.30	43.58 ± 24.51	< 0.001

Values are presented as mean ± SD, or number (%), or median (interquartile range)

*CPC* chest pain centers, *BMI* body mass index, *HR* heart rate, SBP systolic blood pressure, *DBP* diastolic blood pressure, *WBC* white blood cell, *RBC* red blood cell, *ALT* alanine aminotransferase, *AST* aspartate aminotransferase, *CK* creatine kinase, *LDL-C* low-density lipoprotein cholesterol, *HDL-C* high-density lipoprotein cholesterol, *IRA* infarct related artery, *LM* left main coronary artery, *LAD* left anterior descending, *LCX* left circumflex artery, *RCA* right coronary artery, *IABP* intra-aortic balloon pump

In this study, out of 5344 patients, 505 did not undergo coronary angiography. Therefore, coronary artery analysis was conducted on the remaining 4839 patients who underwent coronary angiography or PCI procedures**.** Among the 4839 individuals, the post-CPC group had a higher proportion of three-vessel disease (55.77% vs. 46.41%, *p* < 0.001). The distribution of infarct-related arteries showed no significant differences between the groups, except for a slight decrease in the involvement of the left anterior descending artery (48.10% vs. 52.20%, *p* = 0.006). Additionally, no significant differences were observed between the groups regarding the incidence of no-reflow (4.07% vs. 5.41%, *p* = 0.092) and the use of IABP (3.70% vs. 4.50%, p = 0.349).

Following the establishment of the CPC, significant improvements were observed in patient treatment timelines and quality of care (Table 2). Specifically, the proportion of patients who completed an electrocardiogram (ECG) within 10 min of arrival increased markedly (56.05% vs. 85.73%, *p* < 0.001), and the door-to-balloon (D2B) time was significantly shortened (117.7 ± 117.1 min vs. 46.9 ± 63.4 min, *p* < 0.001). Meanwhile, the rate of primary PCI therapy within 90 min of hospital admission increased substantially (24.47% vs. 60.41%, *p* < 0.001), and the proportion of reperfusion therapy completed within 24 h of symptom onset also improved (53.62% vs. 67.84%, *p* < 0.001).

**Table 2:** Comparison of quality measures for patients with STEMI

	Pre-CPC (n = 2141)	Post-CPC (n = 3203)	*P* value
Completion of ECG within 10 min of arrival	1200 (56.05)	2746 (85.73)	< 0.001
Aspirin at administration within 1 h	1562 (72.96)	2379(74.27)	0.283
P2Y12 receptor inhibitors at administration within 1 h	1517 (70.85)	2380 (74.31)	0.005
Reperfusion therapy within 24 h of symptom onset	1148 (53.62)	2173 (67.84)	< 0.001
Primary PCI therapy within 90 min of hospital admission	524 (24.47)	1935 (60.41)	< 0.001
D2B time (minutes)	117.7 ± 117.1	46.9 ± 63.4	< 0.001
Thrombolysis within 30 min of symptom onset	18 (0.84)	138 (4.31)	< 0.001
Rescue PCI	11(0.51)	98(3.06)	< 0.001
Only medication therapy	244 (11.40)	194 (6.06)	< 0.001
Transfer to CABG	1(0.04)	7(0.22)	0.111
Beta blocker at administration within 24 h	1502 (70.15)	2155 (67.28)	0.027
Evaluation of LVEF during hospitalization	2047 (95.61)	3046 (95.10)	0.216
Aspirin at discharge	1993 (93.09)	3026 (94.5)	0.038
P2Y12 receptor inhibitors at discharge	1995 (93.18)	3021 (94.47)	0.090
Beta-blockers at discharge	1085 (84.30)	2577 (80.46)	< 0.001
ACEI/ARBs at discharge	1331 (62.17)	1785 (55.73)	< 0.001
Statin at discharge	2011 (93.92)	2979 (93.01)	0.184

Values are presented as mean ± SD, or number (%)

*CPC* chest pain centers, *ECG* electrocardiogram, *P2Y12* receptor adenosine diphosphate receptor, *PCI* percutaneous coronary intervention, *D2B* door-to-balloon, *CABG* coronary artery bypass grafting, *LVEF* left ventricular ejection fraction, *ACEI* angiotensin converting enzyme inhibitors, *ARB* angiotensin receptor blocker

**Fig. 2:**
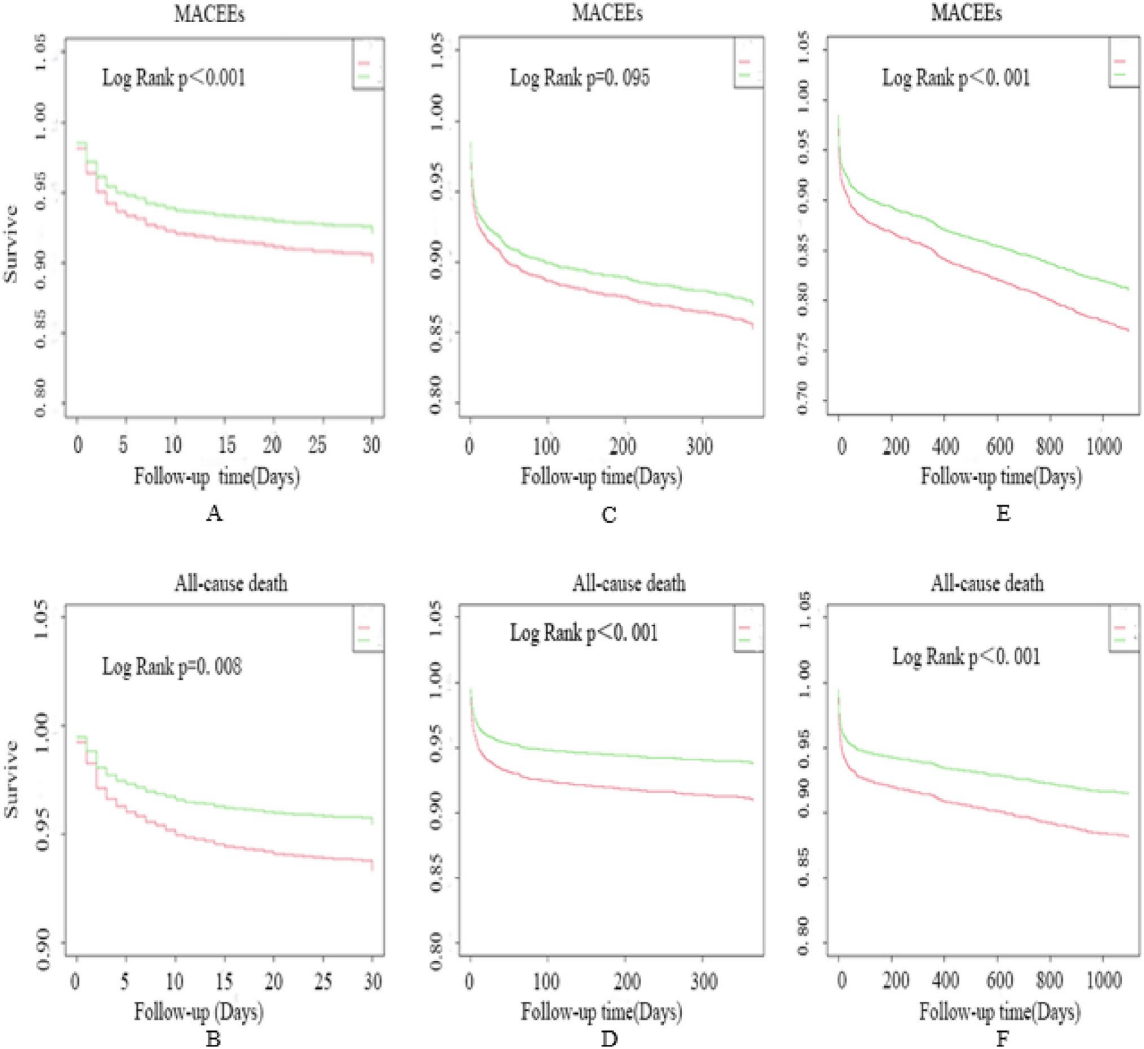
Cumulative Kaplan–Meier curve estimates of outcomes. Data for MACCE (**A**) and all-cause death (**B**) within 30 days; Data for MACCE (**C**) and all-cause death (**D**) within 1 year; Data for MACCE (**E**) and all-cause death (**F**) within 3 year. *MACCE* major adverse cardiovascular and cerebrovascular events

In terms of short- and long-term outcomes (Table 3), the incidence of MACCEs decreased within both 30 days (10.00% vs. 7.90%, *p* = 0.008) and 3 years (23.12% vs. 18.86%, *p* < 0.001). Additionally, the 30-day mortality rate decreased from 6.68% to 4.53% (*p* = 0.001), while the 3-year mortality rate declined from 11.86% to 8.55% (*p* < 0.001), the same can be shown in KM curve (Fig. 2).

**Table 3:** Outcomes within 30 days, 1 year and 3 years after onset*

	30 Days			1 Year			3 Years		
	Pre-CPC (n = 2141)	Post-CPC (n = 3203)	*P* value	Pre-CPC (n = 2141)	Post-CPC (n = 3203)	*P* value	Pre-CPC (n = 2141)	Post-CPC (n = 3203)	*P* value
MACCEs	214 (10.00)	253 (7.90)	0.008	315 (14.71)	421 (13.14)	0.103	495 (23.12)	604 (18.86)	< 0.001
All-cause death	143 (6.68)	145 (4.53)	0.001	195 (9.11)	200 (6.24)	0.001	254 (11.86)	274 (8.55)	< 0.001
Recurrent MI	21 (0.98)	21 (0.66)	0.187	30 (1.40)	53 (1.65)	0.463	63 (2.94)	92 (2.87)	0.881
Recurrent angina	47 (2.29)	61 (1.90)	0.459	84 (3.92)	145 (4.53)	0.286	163 (7.61)	213 (6.65)	0.177
Stroke	23 (1.07)	40 (1.25)	0.562	28 (1.31)	47 (1.47)	0.627	39 (1.82)	64 (2.00)	0.646

Values are presented as mean ± SD, or number (%)

*CPC* chest pain centers, *MACCE* major adverse cardiovascular and cerebrovascular events, *MI* myocardial infarction

^*^Patients may have had > 1 outcome in each category but were counted only once for overall events

**Fig. 3:**
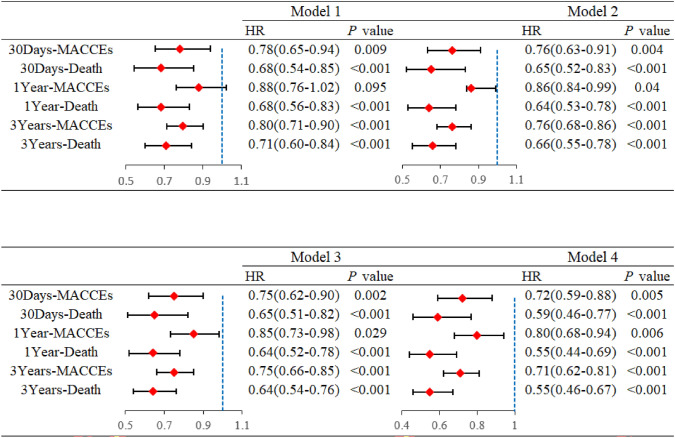
Outcomes within 30 Days, 1 years and 3 years After Onset Associated with CPC Establishment: Unadjusted and Multivariate Adjusted Analyses*. *Model 1 was unadjusted. Model 2: adjusted for age, sex and BMI. Model 3: adjusted age, sex, BMI, diabetes, hypertension, cerebrovascular disease, peripheral vascular disease, atrial fibrillation, chronic obstructive pulmonary, gastrointestinal ulcer history. Model 4: adjusted age, sex, BMI, diabetes, hypertension, cerebrovascular disease, peripheral vascular disease, atrial fibrillation, chronic obstructive pulmonary, gastrointestinal ulcer history, Troponin-T, RBC, Platelet, Albumin, Creatinine, Sodium, Chlorine, Uric acid, ALT, CK, LDL cholesterol, HDL cholesterol, Triglycerides, SBP at admission, DBP at admission, Multivessel disease, Killip classification and No-reflow. *CPC* chest pain centers, *MACCE* major adverse cardiovascular and cerebrovascular events

Cox regression analysis further demonstrated that the establishment of the CPC was associated with a significant reduction in the risk of MACCEs and mortality. Figure 3 highlights the association between Chest Pain Center establishment and improved clinical outcomes in STEMI patients, including reductions in all-cause mortality and major adverse cardiovascular and cerebrovascular events at 30 days, 1 year, and 3 years. The analysis was conducted using four progressively adjusted models to evaluate the impact of CPC implementation on patient outcomes. Model 1, representing baseline unadjusted analyses, demonstrated a significant reduction in MACCEs and all-cause mortality associated with CPC care. Model 2 incorporated basic demographic adjustments, including age, sex, and BMI, highlighting the influence of these characteristics on outcomes. Model 3 further refined the analysis by accounting for comorbidities such as diabetes, hypertension, and peripheral vascular disease, providing a more comprehensive understanding of the factors affecting prognosis. Finally, Model 4, which included adjustments for clinical features such as Killip classification, no-reflow phenomenon, and laboratory biomarkers (e.g., Troponin-T, creatinine, LDL cholesterol), confirmed the robust association between CPC care and improved patient outcomes, emphasizing the multifactorial benefits of this approach.

Across all four models, CPC implementation consistently demonstrated significant benefits. In the fully adjusted model (Model 4), the HR for MACCEs were 0.72 (95% CI 0.59–0.88,* p* = 0.005) at 30 days, 0.80 (95% CI 0.68–0.94), *p* = 0.006 at 1 year, and 0.71 (95% CI 0.62–0.81,* p* < 0.001) at 3 years, while the HRs for all-cause mortality were 0.59 (95% CI 0.46–0.77,* p* < 0.001), 0.55 (95% CI 0.44–0.69,* p* < 0.001), and 0.55 (95% CI 0.46–0.67,* p* < 0.001) over the same timeframes. These findings underscore the substantial and lasting impact of CPC implementation on improving patient outcomes.

## Discussion

This study highlights the significant impact of Chest Pain Centers on improving the diagnosis, treatment, and long-term outcomes of ST-segment elevation myocardial infarction patients in Ningxia, an underdeveloped region in China. CPC implementation reduced 30-day and 3-year mortality rates (6.68–4.53% and 11.86–8.55%, respectively) and MACEEs rates (10.00–7.90% and 23.12–18.86%). These findings underscore the critical role of CPC in addressing disparities in cardiovascular care and enhancing regional healthcare capabilities, especially in resource-limited settings.

In our study, we found CPC establishment as an independent protective factor against both mortality and MACCEs. Adjusted hazard ratios for all-cause mortality were 0.59 at 30 days, 0.55 at 1 year, and 0.55 at 3 years, while HR for MACCEs were 0.72, 0.80, and 0.71 over the same timeframes. Kaplan–Meier survival curves further confirmed the survival benefits, showing significantly higher survival probabilities in the post-CPC group. These findings align with international evidence. In the United States, Ross et al. demonstrated that CPC-accredited hospitals achieve better acute myocardial infarction management outcomes compared to non-accredited hospitals [23]. Similarly, Keller et al. in Europe reported superior 1-year outcomes for acute coronary syndrome (ACS) patients treated in chest pain units compared to traditional emergency departments [24]. In Asia, Alexander et al. showed that a CPC-like model in South India improved PCI utilization and reduced 1 year mortality [25]. Previous studies in China have shown that CPC accreditation is associated with improved in-hospital management and outcomes for ACS and AMI patients [26–28]. However, evidence on the long-term benefits of CPC in economically underdeveloped regions remains limited. The study fills a critical gap by providing robust data on long-term outcomes in Ningxia, demonstrating sustained benefits over a 3 year.

CPC implementation achieved substantial progress in reducing door-to-balloon (D2B) times, from 117.7 min pre-CPC to 46.9 min post-CPC, and increasing the proportion of patients receiving primary PCI within 90 min of hospital admission from 24.47–60.41%. Studies have consistently demonstrated that every 10 min reduction in D2B time significantly lowers the risk of in-hospital mortality [29, 30]. Although these improvements are notable, the overall 24 h reperfusion therapy rate remains lower than in developed countries such as the United States (95% in 2016) and Germany (78% in 2011) [31, 32]. Similarly, the proportion of patients receiving thrombolysis within 30 min of arrival increased from 0.88% pre-CPC to 4.1% post-CPC but still lags behind levels seen in the United States (92–94% in 2010) [33]. Early thrombolytic therapy is crucial for STEMI patients who cannot receive primary PCI promptly, as it can significantly reduce infarct size, improve left ventricular function, and decrease mortality. Evidence suggests that thrombolysis administered within the first 30 min of symptom onset offers the greatest survival benefits, with a 30% reduction in mortality compared to delayed treatment [34, 35]. These delays can be attributed to uneven medical resource distribution, insufficient diagnostic capabilities, and limited training among primary healthcare workers. Addressing these barriers requires targeted interventions, such as strengthening grassroots medical training and promoting telemedicine systems to reduce delays in diagnosis and treatment decisions.

CPC implementation not only refined acute treatment pathways but also fostered adherence to guideline-directed medical therapy (GDMT). Early access to percutaneous coronary intervention and evidence-based pharmacological therapies, such as dual antiplatelet therapy and high-intensity statins, are fundamental aspects of CPC protocols [8, 36]. GDMT has been shown to play a pivotal role in reducing major adverse cardiac and cerebrovascular event rates and improving survival outcomes in STEMI patients. For instance, the use of dual antiplatelet therapy is associated with a significant reduction in ischemic events and stent thrombosis, while high-intensity statins reduce recurrent cardiovascular events and improve overall mortality [37, 38]. This study observed improved use of beta-blockers within 24 h and P2Y12 receptor inhibitors at discharge, reflecting enhanced adherence to evidence-based practices. Beta-blockers are critical for reducing myocardial oxygen demand, limiting infarct size, and improving long-term outcomes, particularly when administered early [39]. Similarly, timely initiation of P2Y12 receptor inhibitors has been demonstrated to lower platelet reactivity and reduce thrombotic complications, contributing to better prognosis in STEMI patients [40]. These interventions collectively highlight the importance of incorporating GDMT into routine CPC protocols, which are essential for reducing MACCEs rates and improving long-term survival.

### Challenges in CPC implementation in resource-limited regions

Despite the substantial contributions of Chest Pain Centers (CPCs), their implementation in underdeveloped regions faces several persistent challenges that must be addressed to maximize their effectiveness. One of the foremost obstacles is economic constraint. Establishing and maintaining CPCs demands considerable financial investment, including infrastructure upgrades, equipment procurement, and personnel training. In low-resource settings, many hospitals struggle with budget limitations, hindering the long-term sustainability of CPC operations [25]. To overcome this, policymakers should consider innovative funding strategies such as government subsidies, public–private partnerships, and performance-based incentive programs [41].

In addition to financial barriers, a shortage of skilled healthcare professionals poses a critical challenge. Effective STEMI care within CPCs depends on the coordinated work of emergency physicians, cardiologists, nurses, and emergency medical service (EMS) providers. However, economically disadvantaged regions often suffer from workforce shortages and high staff turnover. Addressing this issue requires the implementation of standardized CPC-specific training and ongoing continuing medical education (CME) programs to improve healthcare providers’ competencies in diagnosis, risk stratification, and timely reperfusion therapy.

Furthermore, limited public awareness and pre-hospital delays significantly hinder early STEMI management. Many patients in rural and underserved areas are unaware of heart attack symptoms, lack access to emergency transportation, or rely on local clinics and self-medication, which leads to delayed hospital arrival. Public health campaigns that educate communities about symptom recognition, emphasize the urgency of contacting emergency services, and promote the use of pre-hospital ECG screening are essential to reduce delays and improve triage efficiency.

By addressing these interconnected barriers, CPCs can be more effectively integrated into underdeveloped healthcare systems, ultimately enhancing equity and outcomes in acute cardiac care.

### Policy-level recommendations for CPC expansion

To ensure the sustainability and scalability of Chest Pain Centers (CPCs) in resource-limited regions, a coordinated set of policy-level strategies is essential. Government leadership should play a central role by prioritizing CPC development in underserved areas. This includes allocating targeted funding, incorporating CPCs into national STEMI care networks, and establishing region-specific accreditation standards to guide implementation and quality assurance.

Equally important is the strengthening of regional STEMI networks through a hub-and-spoke model. In this approach, tertiary hospitals function as central referral hubs, supporting smaller, rural healthcare facilities in optimizing patient transfer pathways and resource allocation. This structure fosters a more coordinated and efficient continuum of care, particularly in geographically dispersed or low-resource settings. The integration of telemedicine and artificial intelligence (AI)-based decision support also holds great promise. Expanding telemedicine capabilities within CPCs can enhance real-time remote electrocardiogram (ECG) interpretation, support earlier STEMI identification, and guide pre-hospital triage decisions. Moreover, AI-driven risk prediction tools may improve clinical decision-making by enabling more accurate and individualized treatment strategies [42].

By systematically addressing the financial, workforce, and infrastructure-related barriers, these approaches can significantly enhance the reach and impact of CPCs. Ensuring timely and equitable STEMI care in underdeveloped areas not only improves clinical outcomes but also strengthens the broader healthcare system. Future research should aim to evaluate the effectiveness of these strategies and identify scalable best practices to guide CPC implementation in resource-constrained environments.

This study offers valuable insights to both cardiovascular medicine and health systems research. First, this study stands out as one of the initial pieces of evidence showcasing the long-term benefits of CPC establishment on STEMI outcomes in a low- to middle-income country (LMIC). This expands the current comprehension of CPC effectiveness, which has primarily been studied in high-income settings. Second, the study uses a large, real-world dataset from the Ningxia Myocardial Infarction Registry, ensuring robust external validity and reflecting the realities of clinical practice in China. Third, this study highlights the crucial role of collaborative, multi-faceted healthcare models for vulnerable populations. By demonstrating sustained reductions in mortality and MACCE rates over a 3 year period, the study highlights the value of CPCs not only in acute care but also in long-term secondary prevention. These results contribute to the growing evidence base supporting health system reforms aimed at optimizing STEMI care in resource-limited settings. Finally, this study bridges a critical evidence gap by quantifying the long-term survival benefits and event-free outcomes associated with CPCs, providing crucial insights for policymakers and healthcare administrators.

This study has several limitations that warrant consideration. First, due to its retrospective cohort design, causal inferences cannot be firmly established, despite adjustments for multiple confounding factors via multivariable Cox regression. Despite acknowledging the potential impact of baseline differences on our study outcomes, we choose to employ multivariable Cox regression rather than Propensity Score Matching (PSM) to address this issue. While PSM remains a viable method, it has the potential to decrease statistical power due to unmatched cases and may introduce bias if matching is not fully achieved [43, 44]. Future studies may consider incorporating PSM or more advanced analytical techniques such as time-series or multilevel modeling to further validate our findings and better isolate the effect of CPC implementation. Second, the study was conducted at a single center, which may limit the generalizability of the results to other regions in China with varying healthcare infrastructures. Multicenter studies are needed to explore regional differences and confirm the broader applicability of CPC-related improvements. Third, while the study focused on long-term mortality and MACCEs, it did not include other important outcomes such as healthcare costs, resource utilization, or health-related quality of life. Although we observed reduced hospital stays and costs in the post-CPC group, a formal cost-effectiveness analysis was not performed. Moreover, patient-reported outcomes and quality-of-life metrics were not captured, despite their growing recognition as essential endpoints in cardiovascular research. Fourth, our registry did not systematically collect data on medication adherence, participation in secondary prevention programs, or bleeding complications. In particular, major hemorrhagic events-which are critical to assess in STEMI patients receiving antithrombotic therapy-were incompletely recorded. Future research should incorporate standardized bleeding assessments, such as those defined by the Bleeding Academic Research Consortium (BARC), and track adherence through real-world data sources (e.g., pharmacy records or follow-up surveys). Lastly, while CPC implementation was temporally associated with improved outcomes, other concurrent improvements in the healthcare system—such as enhanced access to interventional services, availability of new medications, and better provider training-may also have contributed. Although baseline characteristics were generally balanced between groups, we did not systematically capture changes in healthcare infrastructure over time, leaving residual confounding possible.

The findings of this study strongly advocate for the expansion of CPC networks across China to standardize care for STEMI patients. Policymakers should prioritize investments in CPC and incentivize hospitals to adopt evidence-based protocols. Public awareness campaigns and training programs for healthcare professionals are essential to maximize the impact of CPC, particularly in underserved areas. Moreover, leveraging digital health tools, such as telemedicine and artificial intelligence, could further enhance the efficiency and accessibility of CPC systems.

## Conclusion

This study demonstrates that the establishment of Chest Pain Centers (CPCs) significantly improves the long-term prognosis of patients with acute ST-segment elevation myocardial infarction (STEMI) in China. The findings reveal consistent reductions in 30 day, 1 year, and 3 year mortality, as well as decreased rates of major adverse cardiovascular and cerebrovascular events (MACCEs). These benefits are likely driven by enhanced adherence to evidence-based treatment protocols, expedited reperfusion therapy, and overall improvements in care quality. In addition to clinical gains, our findings suggest that CPC implementation may reduce healthcare costs. Patients in the post-CPC group experienced shorter hospital stays and lower in-hospital expenditures, reflecting improved treatment efficiency. Furthermore, standardized care and timely intervention may help reduce long-term complications and rehospitalization risk, offering potential economic value.

Together, these results underscore the critical importance of expanding CPC networks nationwide to optimize STEMI management and patient outcomes. Future research should aim to reduce regional disparities in CPC access, improve public awareness, and integrate advanced technologies such as telemedicine and AI to maximize the impact of CPCs on survival and healthcare sustainability.

## Data Availability

The datasets generated and/or analyzed during the current study are not publicly available due to the ongoing nature of this study but are available from the corresponding author on reasonable request.
